# Spanish version of the Parkinson’s Disease Questionnaire–Carer (PDQ-Carer)

**DOI:** 10.1186/s12955-016-0546-z

**Published:** 2016-11-04

**Authors:** Rosario Ferrer-Cascales, María José Cabañero-Martínez, Miriam Sánchez-SanSegundo, Nereida Congost-Maestre, Crispin Jenkinson

**Affiliations:** 1 Department of Health Psychology, Faculty of Health Science, University of Alicante, Alicante, Spain; 2 Department of Nursing, University of Alicante, Alicante, Spain; 3 Department of English Philology, University of Alicante, Alicante, Spain; 4 Nuffield Department of Population Health, University of Oxford, Oxford, UK

**Keywords:** Caregivers, Parkinson disease, PDQ-Carer, Psychometric properties, Quality of life

## Abstract

**Background:**

Parkinson’s caregivers are frequently affected by a range of physical and psychological factors affecting to the quality of life (QoL) of patients and caregivers. However, while there are well-validated QoL instruments for patients, few specific measures has been developed for caregivers of patients with PD. This study examined the psychometric properties of the Spanish version of the Parkinson Disease Questionnaire–Carer (PDQ-Carer) for use in PD caregivers.

**Methods:**

The PDQ-Carer and the Short Form-36 Health Survey (SF-36) were completed by sample of 73 caregivers of patients with PD in Spain (71.8 % females; 63.6 ± 12.3 years old).

**Results:**

Psychometric analysis confirmed the reliability and validity of the Spanish version of the PDQ-Carer. The internal consistency was found to be satisfactory for the four PDQ-Carer domains: Personal and Social Activities, Depression and Anxiety, Self-care and Stress with Cronbach’s alpha values ranging 0.80 to 0.95. The PDQ-Carer was significantly correlated with the eight SF-36 domains (*r* = -0.31 to -0.59, *p* < 0.001) supporting the concurrent validity of the instrument.

**Conclusions:**

Overall, these results provide preliminary evidence of the utility of the Spanish version of the PDQ-Carer in non-professionals caregivers

## Background

Parkinson’s disease (PD) is a chronic and progressive neurodegenerative disorder characterised by physical and psychological symptoms that result in motor disability, neuropsychiatric symptoms and loss of autonomy [1, 2]. PD is the second most common neurodegenerative disorder after Alzheimer’s disease affecting approximately 1-2 % of the population over 60 years of age and 4 % of those aged 80 years worldwide [3]. According to disease prevalence projections, it is estimated that the number of patients with Parkinson’s disease in Western Europe will grow from 4 million in 2005 to 9 million in 2030 [4]. In Spain, around 6.400 individuals are affected by Parkinson’s disease each year and about 30 % of cases are diagnosed in advanced stages of the disease [5, 6]. This figure is expected to increase due to the growing elderly population, particularly in the group of individuals aged 70 and older [7].

As a consequence of the progressive nature of the disease most patients with PD are affected by a range of cognitive and behavioural disorders which are observed even in the early stages of the disease, as a result of the loss of dopaminergic neurons and the progressive decrease of the activity in several areas of the brain [8]. These cognitive deficits are often accompanied by clinical symptoms such as bradykinesia, rigidity and resting tremor, which has been reported to be prognostic of the progressive course of the disease, [9] making difficult for people with Parkinson to carry out basic and instrumental everyday activities without the assistance of family members and other non-informal caregivers [10]. Parkinson’s caregivers have been recognized to play an important role in supporting patients, particularly in advanced stages of the disease leading a reduction of the rates of institutionalization and number of hospitalizations [10, 11]. However, caring for a family member with PD has been considered as a stressful process that affects negatively physical, psychological and socioeconomic conditions of caregivers [12]. It has been reported that caring a patients with PD is strongly related to poor psychosocial outcomes including high levels of burden, depression, anxiety, mood disturbances and emotional distress [13–15]. These common manifestations that affect the quality of life (QoL) of carers of individuals with PD are frequently associated with the severity of symptoms of patients, progression disease and patient disability [14]. Several studies examining the role of caregiving in Spain and other Western European countries have found that patient disability and mood disturbances contribute to caregiver’s burden and stress as well as placing strain on the family unit as a whole [1, 16, 17]. In addition, the demands of caring may result in an increase in marital dissatisfaction since caregiving may result in a lack of leisure activities, social isolation and low levels of perceived social support [18]. Given that the QoL of PD caregivers can be adversely affected by a range of factors, it is important to assess the areas of greatest impairment associated to role of caregiver. Awareness of the impact of PD on carers may enable effective interventions aimed at improving caregiver’s quality of life of patients and ameliorating the negative effects of caregiving [15].

Previous research has often measured QoL in carers of patients with Parkinson’s disease by using a variety of generic scales including the Zarit Caregiver Burden Inventory [19] and the World Health Organisation Quality of Life Scale (WHOQoL) [20] which were originally developed for caregivers of patients with dementia [21]. More recently several instruments such as the BELA-A-K [12] and the Scale of Quality of Life Care-Givers (SQLC) [22] have been specifically developed for caregivers of people with PD. Although these measures provide clinically important information of physical and psychological factors affecting to the role of caregivers, their feasibility has not formally been reported for English and Spanish speaking population [23]. In addition, the Scale of Quality of Life Care-Givers (SQLC) has been subject to criticism due to the complexity of both administration and scoring [23].

Recently, a short and easy to administrate health related quality of life questionnaire for use with carers of patients with PD have been developed in UK. The Parkinson’s Disease Questionnaire fore Carers (PDQ-Carer) attempts to quantify subjective experiences of caregivers by examining the main important domains that are adversely affected by the role of caring: Social and Personal Activities, Anxiety and Depression, Self-Care and Stress [23]. The PDQ-Carer has been shown to be valid, reliable and sensitive to change for English population. However, cross-cultural validation has not been reported in other cultures. Therefore, the purpose of this study was to examine the psychometric properties of the Spanish version of the PDQ-Carer. We examined the PDQ-Carer in terms of reliability and validity by examining a sample of 73 caregivers of patients with PD.

## Methods

### Participants

Participants were recruited from four Parkinson’s Associations located in the Spanish regions of Alicante, Albacete, Pontevedra and Tarragona. The contact with these Associations was carried out by a senior researcher through the Organización Unidos Contra el Parkinson (http://portal.unidoscontraelparkinson.com/). Letters of invitation were sent to the associations to participate in the research after being informed of the purpose of the study. The questionnaires were sent to the participant associations by mail who then distributed these to members. The questionnaire was self-administered by participants. The recruitment period was conducted between January 2015 to June 2015. A total of 105 participants were approached to take part of whom 73 (69.5 %) agreed to participate while 32 (30.5 %) declined. Caregiver was defined according to Martínez-Martín [13] as “any relative or person who is not a professional caregiver or member of social support network, usually living with the patient and directly involved in caring the patient or directly affected by the patient’s health problem”. Caregivers were included if they were i) over 18 years of age, ii) able to speak, read and write into Spanish, iii) able to give their informal consent. Exclusion criteria included the i) absence of a clearly identified caregiver, ii) professional and paid caregivers and iii) presence of other problems which would not allow them to answer the questionnaire (such as psychiatric problems, neurologic diseases and learning disabilities). Full informed consent was obtained from each participant prior to participation after receiving complete information on the study. The study was approved by the Ethics Committee from different Associations of PD and it was conducted according to the Ethical Principles for Medical Research Involving Human Subjects [24].

### Measures

#### The Parkinson’s Disease Questionnaire for Carers (PDQ-Carer)

The PDQ-Carer [23, 25] is designed to assess quality of life related with the health of people who care for relatives with Parkinson’s Disease. The PDQ-Carer contains 29 questions divided into 4 dimensions that evaluate: social and personal activities (12 items); anxiety and depression (6 items); strain (6 items); and self-care (5 items). Questions are scored using a Likert scale from 0 to 4, with the following response options: (0 = never, 1 = almost never, 2 = sometimes, 3 = almost always, 4 = always). The PDQ-Carer provides a global score for each of the dimensions, where 0 indicates a complete absence of problems, and 100 the maximum level of problems. The score is calculated as follows: scale score equals the total of the raw scores of each item in the scale divided by the maximum possible raw score of all the items in the scale multiplied by 100. In general, higher scores indicate lower QoL. The psychometric properties of the original version of the instrument have proven to be good, with Cronbach’s alpha coefficients of 0.85 or above in all four scales [23].

#### The Short Form-36 Heath Survey (SF-36)

The SF-36 [26] is a self-administrate generic health related HR-QoL questionnaire which has been extensively validated in a wide range of population. The measure is composed of 36 questions and standardized response choices. It provides scores on eight dimensions: Physical Functioning, Role Physical (limitations due to physical health problems), Role Emotional (limitations due to emotional problems), Social function, Vitality, Pain, Mental Health, and General Health Perceptions. Scores for each dimension range from 0 (poor health) to 100 (good health), where higher scores indicate better QoL. The Spanish version of the SF-36 has been extensively used, and shown to have excellent psychometrics properties [27].

#### Sociodemographic and clinical variables

Sociodemographic data of caregivers (gender, age, marital status, level of education, relationship with the patient, type of carer, time as a carer, working situation, and type of care provided) were prospectively collected. In addition, we examined the following PD patient’s variables: sex, age, and years of disease. The physical stage of disease was self-reported by caregivers based on the medical diagnosis. In addition, the extent of caring was measured by using the following questions: Have you had to leave your job to look after your relative? Do you live with the relative you care for? How many hours do you dedicate to caring for your relative?

### Procedure

#### Transcultural adaptation

The process of translating and validating *The Parkinson’s Disease Questionnaire for Carers* (PDQ-Carer) was conducted in accordance with the guidelines provided by iOutcomes, the copyright holder of the measure, and a wholly owned subsidiary of the University of Oxford. The methodology used by iOutcomes follows the directives of ISPOR [28]. When translating this instrument, two direct translations were provided by two Spanish translators, along with two back translations carried out by native English speakers. Both groups of translators evaluated the difficulty of translating each of the items independently, scoring them on a scale of linguistic-cultural adaptation from 0 (no difficulty) to 10 (maximum difficulty). They were also asked to indicate the types of changes they needed to make during the translation process: A (no changes and same syntactical structure); B (changes required to syntax or semantics and/or cultural expressions); or C (if the item is not applicable to the target cultural context).

#### Pilot testing of interpretability

In order to examine the interpretability of the questionnaire, cognitive interviews were carried out in a pilot sample. Participants were selected based on their gender and stages of the disease among the users of the Alicante Parkinson Association. The pilot version of the questionnaire was administered to 8 PD caregivers of patients with PD, 4 (50.0 %) male and 4 (50.0 %) female, with an average of age of 68.7 (*SD* = 8.4; range (54-78). Evaluations were carried out by two health psychologists and one linguist who specialises in the language of health sciences.

### Statistical analysis

The size of the study group was obtained from previous validation studies into other languages, stipulating that it should contain at least 50 patients [29]. Firstly, a descriptive analysis was conducted of the items. The feasibility of the Spanish version was determined by analyzing the distribution of scores, and the frequency of the maximum and minimum values registered for each of the dimensions. To evaluate internal consistency, Cronbach’s alpha coefficient was calculated for each of the dimensions, along with the item-test correlation for each dimension, and the corrected alpha for each of the items on the questionnaire. Nunnally and Bernstein proposed a criterion of 0.70-0.90 as a measure of good internal consistency, and this is the most widespread criteria used to interpret Cronbach’s alpha coefficients [30–32]. To examine construct validity, the association of each of the scales on the PDQ-Carer with the dimensions on the SF-36 was evaluated using Spearman’s rho correlation coefficient. According to Salkind [33] correlations are considered very strong when greater than 0.8, strong between 0.6 and 0.8, moderate between 0.4 and 0.6, between 0.2 and 0.4 weak and less than 0.2 very weak. Statistical analyses were carried out with the statistical software package SPSS, version 22.0.

## Results

### Descriptive characteristics of the sample

Of the 73 carers (Table 1) who completed the questionnaire, the average age was 63.3 years (SD: 12.3; *n* = 61). The majority of caregivers were women (71.8 %; *n* = 51). 95.9 % were married (*n* = 70), the majority were the patient’s spouse (82.2 %; *n* = 60), and 89.0 % lived with their dependent relative (*n* = 65). The majority had a primary school (49.3 %; *n* = 36) and were retired (41.1 %; *n* = 30) or were homemakers (34.2 %; *n* = 25) and 12.5 % (*n* = 9) had been forced to leave their job to take care of their relative. Most of the participants (81.4 %; *n* = 57) were the patient’s main caregiver, and 53.7 % (*n* = 36) had been caring for their relative for 5 or more years.

**Table 1:** Sociodemographic characteristics of carers and patients

	Mean (SD)	% (n)
Characteristics of the carer		
Age of carer (*n* = 61)	63.3 (12.3)	
Age of patient (*n* = 69)	69.4 (8.2)	
Gender of carer (*n* = 71)		
Male		28.2 (20)
Female		71.8 (51)
Gender of patient (*n* = 72)		
Male		65.3 (47)
Female		34.7 (25)
Marital status of carer (*n* = 73)		
Single		4.1 (3)
Marriage		95.9 (70)
Family relationship between carer and patient (*n* = 73)		
Son/Daughter		11.0 (8)
Spouse		82.2 (60)
Brother/Sister		1.4 (1)
Nephew/Niece		2.7 (2)
Grandson/Granddaughter		1.4 (1)
Not related		1.4 (1)
Lives with patient (*n* = 73)		
Yes		89.0 (65)
No		11.0 (8)
Carer’s level of education (*n* = 73)		
No education		6.8 (5)
Primary Education		49.3 (36)
Secondary Education		27.4 (20)
Higher Education		16.4 (12)
Working situation of carer (*n* = 73)		
Employed part time		5.5 (4)
Employed full time		13.7 (10)
Unemployed		5.5 (4)
Retired		41.1 (30)
Homemaker		34.2 (25)
Left job to care for relative (*n* = 72)		
Yes		12.5 (9)
No		87.5 (63)
Type of carer (*n* = 70)		
Primary caregiver		81.4 (57)
Care shared with other relatives		14.3 (10)
Care shared with contracted staff		4.3 (3)
Years as a carer (*n* = 67)		
1 year or less		10.5 (7)
2 years		11.9 (8)
3 years		14.9 (10)
4 years		9.0 (6)
5 years or more		53.7 (36)
Minimum type of care provided (multiple responses)		
Personal care		65.0 (39)
Housework		53.3 (32)
Transport		51.7 (31)
Administrative and financial issues		51.7 (31)
Stage of the disease (*n* = 71)		
Mild		16.9 (12)
Moderate		49.3 (35)
Severe		33.8 (24)
Length of time living with disease (*n* = 72)		
1 year		5.6 (4)
2 years		13.9 (10)
3 years		15.3 (11)
4 years		6.9 (5)
5 years or more		58.3 (42)

As seen in Table 1, patients had an average age of 69.4 years (SD: 8.2; *n* = 69). 65.3 % (*n* = 47) were male. Most patients were in a moderate stage of the disease (49.3 %; *n* = 35), 33.8 % (*n* = 24) were severe, and in all the other cases it was mild. Around the half of patients had had the disease for 5 or more years (58.3 %; *n* = 42), and 65.0 % of patients required help with personal care (*n* = 39).

### Transcultural adaptation

In the processes of translation and back translation, the translators rated the translation of the items overall as presenting a medium-low level of difficulty, with an average of 3.24 (*SD* = 1.67). The items that were felt to present a greater level of difficulty were numbers 7 (*M* = 4.75; *SD* = 0.96) and 19 (*M* = 4.75; *SD* = 2.06), and those that generated the least difficulty were 13 (*M* = 1.75; *SD* = 0.96) and 15 (*M* = 1.50; *SD* = 1.00). The changes made were of type B in 62.07 % of cases (*n* = 18), whereas 37.93 % of cases (*n* = 11) only required type A changes (Table 2).

**Table 2:** Mean of difficulty and type of change in the cross-cultural process

Items of the PDQ-Carer	Mean (dt) difficulty	Type of change
Item 7. Thought that your caring role was taken for granted by others?	4.75 (0.96)	B
Item 19. Felt that your workload around the house has increased significantly?	4.75 (2.06)	B
Item 16. Felt less in control of your temper than before you became a carer?	4.50 (2.08)	B
Item 3. Found the demands of caring physically difficult?	4.25 (2.75)	B
Item 4. Felt anxious because of the responsibility of caring?	4.00 (2.94)	B
Item 14. Felt more withdrawn because of your caring role?	4.00 (0.82)	B
Item 5. Been prevented from pursuing hobbies and other interests?	3.75 (0.96)	A
Item 18. Been limited in what you can do socially?	3.75 (1.26)	B
Item 24. Felt that you cannot do things on the spur of the moment?	3.75 (2.22)	B
Item 28. Felt responsible for Parkinson’s disease medication being available and taken at appropriate times?	3.75 (2.76)	B
Item 29. Had to limit outings because you worry that the person you care for won’t be able to cope?	3.75 (2.50)	B
Item 25. Found it difficult to be involved in regular activities which require commitment, e. g. volunteering work, regularly meeting friends?	3.50 (2.38)	B
Item 17. Felt worried about what would happen if you were unwell?	3.25 (2.06)	A
Item 20. Found it difficult to see friends and family?	3.25 (2.06)	B
Item 22. Felt that your physical health has been affected by your caring role?	3.25 (1.89)	B
Item 8. Felt that relationships with friends have been affected?	3.00 (1.83)	B
Item 12. Felt you lacked the energy and motivation to do the things you enjoy?	3.00 (2.16)	B
Item 21. Found it difficult to leave the person you care for alone for more than one hour?	3.00 (2.16)	B
Item 26. Paid less attention to your own health (e.g. put off visiting a doctor, ignored symptoms etc)?	3.00 (2.16)	A
Item 27. Felt unable to go on holiday or take short breaks?	3.00 (1.82)	A
Item 2. Found it difficult to get out, for example, to do the shopping?	2.75 (2.06)	B
Item 6. Felt worried about your own physical health?	2.75 (2.06)	A
Item 23. Felt that you are responsible for everything at home?	2.75 (2.06)	A
Item 9. Felt impatient with the person you care for?	2.50 (1.73)	B
Item 10. Felt exhausted?	2.50 (1.29)	A
Item 11. Felt worried about the future?	2.25 (1.50)	A
Item 1. Found you could not sleep through the night?	2.00 (1.41)	A
Item 13. Taken less care with your diet?	1.75 (0.96)	A
Item 15. Felt depressed?	1.50 (1.00)	A

A: no changes and same syntactical structure; B: changes required to syntax or semantics and/or cultural expressions

As for the cognitive interviews carried out, in general there were very few comprehension problems. Only 5 items were found to have any comprehension problem (items 3, 7, 25, 26, 28). Problems were solved by adding syntactical and lexical changes. On the basis of the data obtained, the main team of researchers revised the pilot version and, having agreed on the proposed modifications, the process was closed, giving rise to the definitive Spanish version of the PDQ-Carer. There were no statistically significant differences with the total sample in age (T-Student = 1.22; *p* = 0.225), gender (*X* ^2^ = 0.36; gl = 1; *p* = 0.850) nor stage of disease of the patient (*F* = 0.65; *p* = 0.421), but caregivers differed in educational level (*F* = 3.37; *p* = 0.07).

### Performance of PDQ-Carer

Descriptive statistics for the 3 main results are reported in Table 3. The average score on the PDQ-Carer were 44.15 (*SD* = 28.82) for the Personal and Social Activities Scale, 46.86 (*SD* = 22.97) for the Anxiety and Depression Scale, 32.53 (*SD* = 26.10) for the Self Care Scale, and 48.17 (*SD* = 22.31) for Strain. Of these, only the Self Care Scale presented a slight floor effect.

**Table 3:** Descriptive statistics of PDQ-Carer and SF-36 (*n* = 73)

	Mean	SD	Range	n (%) high scores	n (%) low scores
PDQ-Carer: Personal and Social Activities	44.15	28.82	0-95.83	4 (5.5)	1 (1.4)
PDQ-Carer: Anxiety and Depression	46.86	22.97	0-100	2 (2.7)	1 (1.4)
PDQ-Carer: Self Care	32.53	26.10	0-90.0	12 (16.4)	2 (2.7)
PDQ-Carer: Strain	48.17	22.31	0-91.67	3 (4.1)	1 (1.4)
SF-36: Physical Functioning	71.51	27.81	5.00-100	1 (1.4)	12 (16.4)
SF-36: Role Physical	71.92	39.07	0-100	11 (15.1)	43 (58.9)
SF-36: Role Emotional	64.84	44.41	0-100	20 (27.4)	42 (57.5)
SF-36: Social Functioning	67.12	27.68	0-100	1 (1.4)	18 (24.7)
SF-36: Mental Health	62.63	23.93	0-100	1 (1.4)	3 (4.1)
SF-36: Energy/Vitality	54.18	23.50	0-100	1 (1.4)	2 (2.7)
SF-36: Pain	60.22	28.44	0-100	1 (1.4)	14 (19.2)
SF-36: General Health Perception	56.34	20.84	5.00-97.00	1 (1.4)	1 (1.4)

### Reliability

Internal consistency of scales was assessed and found Cronbach’s Alpha coefficients of 0.95 for the Personal and Social Activities scale, 0.85 for the Anxiety and Depression and Self Care scales, and 0.80 for the Strain scale. The Alpha coefficient value from the Strain scale was slightly increased after removing item 7.

Item-total correlations for the Personal and Social Activities scale yielded values of between 0.90 for item 25 and 0.63 for item 24. For the Anxiety and Depression scale, correlations oscillated between 0.61 for items 6 and 17, and 0.69 for item 4. In the case of the Self Care scale, the values of the correlations ranged from 0.51 for item 13 and 0.74 for item 3. The Strain scale yielded the lowest correlation once again for item 7 (0.33), with the highest correlation being 0.81 for item 10 with the total scale (Table 4).

**Table 4:** Item to total correlations and internal reliability consistency of PDQ-Carer Scales

Items of the PDQ-Carer	Cronbach’s alpha	Corrected alpha	Item-test
Personal and Social Activities (12 items)	0.95		
Item 5. Been prevented from pursuing hobbies and other interests?		0.94	0.80
Item 8. Felt that relationships with friends have been affected?		0.94	0.79
Item 14. Felt more withdrawn because of your caring role?		0.95	0.73
Item 18. Been limited in what you can do socially?		0.94	0.80
Item 19. Felt that your workload around the house has increased significantly?		0.95	0.77
Item 20. Found it difficult to see friends and family?		0.94	0.86
Item 21. Found it difficult to leave the person you care for alone for more than one hour?		0.95	0.74
Item 24. Felt that you cannot do things on the spur of the moment?		0.95	0.63
Item 25. Found it difficult to be involved in regular activities which require commitment, e. g. volunteering work, regularly meeting friends?		0.94	0.90
Item 27. Felt unable to go on holiday or take short breaks?		0.95	0.74
Item 28. Felt responsible for Parkinson’s disease medication being available and taken at appropriate times?		0.95	0.69
Item 29. Had to limit outings because you worry that the person you care for won’t be able to cope?		0.95	0.77
Anxiety and Depression (6 items)	0.85		
Item 4. Felt anxious because of the responsibility of caring?		0.82	0.69
Item 6. Felt worried about your own physical health?		0.83	0.61
Item 11. Felt worried about the future?		0.82	0.65
Item 12. Felt you lacked the energy and motivation to do the things you enjoy?		0.82	0.67
Item 15. Felt depressed?		0.83	0.62
Item 17. Felt worried about what would happen if you were unwell?		0.83	0.61
Sef Care (5 items)	0.85		
Item 2. Found it difficult to get out, for example, to do the shopping?		0.82	0.67
Item 3. Found the demands of caring physically difficult?		0.80	0.74
Item 13. Taken less care with your diet?		0.86	0.51
Item 22. Felt that your physical health has been affected by your caring role?		0.81	0.73
Item 26. Paid less attention to your own health (e.g. put off visiting a doctor, ignored symptoms etc)?		0.82	0.69
Strain (6 items)	0.80		
Item 1. Found you could not sleep through the night?		0.79	0.48
Item 7. Thought that your caring role was taken for granted by others?		0.84	0.33
Item 9. Felt impatient with the person you care for?		0.76	0.63
Item 10. Felt exhausted?		0.72	0.81
Item 16. Felt less in control of your temper than before you became a carer?		0.76	0.64
Item 23. Felt that you are responsible for everything at home?		0.76	0.61

### Validity

Construct validity was examined by means of correlations of scales PDQ-Carer with scales for SF-36. Personal and Social Activities (PDQ-Carer) obtained moderate correlation with all SF-36 scales, the higher correlation was - 0.59 (*p* < 0.001) with Social Functioning. The higher correlation of the Anxiety and Depression (PDQ-Carer) scale was -0.58 (*p* < 0.001) with Mental Health scale of the SF-36. Self-care scale (PDQ-Carer) was correlate with all of SF-36 scales with correlate values between -0.51 (*p* < 0.001) with Role Physical and -0.58 (*p* < 0.001) with Social Functioning. Finally, Carer Strain scale (PDQ-Carer) obtained moderate correlation with Mental Health (rho = -0.53; *p* < 0.001), Vitality (rho = -0.52; *p* < 0.001) and Social Functioning (-0.49; *p* < 0.001). Correlations between SF-36 and PDQ-Carer scales are negative due to the inverse scoring algorithms of the measures (Table 5).

**Table 5:** Correlations (Rho Spearman) between PDQ-Carer and SF-36

	PF	RP	RE	SF	MH	E/V	PAIN	GHP
PDQ-Carer: Personal and Social Activities	-0.42	-0.43	-0.45	-0.59	-0.53	-0.50	-0.40	-0.46
PDQ-Carer: Anxiety and Depression	-0.45	-0.37	-0.50	-0.45	-0.58	-0.53	-0.44	-0.59
PDQ-Carer: Self Care	-0.54	-0.51	-0.54	-0.58	-0.52	-0.55	-0.49	-0.57
PDQ-Carer: Strain	-0.31	-0.35	-0.44	-0.49	-0.53	-0.52	-0.42	-0.43

Note: SF-36: *PF* Physical Functioning, SF-36: *RP* Role Physical, SF-36: *RE* Role Emotional, SF-36: *SF* Social Functioning, SF-36: *MH* Mental Health, SF-36: *E/V* Energy/Vitality, SF-36: *GHP* General Health Perception. All correlations significant at 0.001. The negative correlations are due to inverse scoring algoithms on the SF-36 and PDQ-Carer

## Discussion

This is one the first studies in Spain to examine the relative contributions of health related quality of life in caregivers of patients with Parkinson’s Disease. The aim of the current study was to examine the psychometric properties of the Spanish version of the PDQ-Carer, a new quality of life measure specifically developed for Parkinson’s caregivers. The preliminary findings of this study suggest that the PDQ-Carer Spanish version shows good psychometric properties in terms of cross-cultural adaptation, reliability and validity comparable to those reported in the original English version [23]. Overall, the PDQ-Carer Spanish version was found to be easy to understand for respondents and few linguistics changes were required after the first stage of the cross-cultural validation into Spanish.

In terms of reliability, both the item-total correlation and Cronbach’s alpha values were found to be satisfactory for the four domains measured by the PDQ-Carer (Personal and Social Activities, Anxiety and Depression, Self-care and Strain). The alpha coefficient values ranged from 0.80 to 0.95, comparable to those found by Jenkinson et al., [23] in the original development and validation of this measure. The most significant alpha scores were related to personal and social activities scale, which examines how caring for a patient with PD may affect relationships with others as well as the ability to maintain pastimes and hobbies. Studies examining the relationship between activity level, wellbeing and quality of life among caregivers of patients with PD, have demonstrated that a loss of activity level as a consequence of caring might lead to a decrease in quality of life and contribute to a major impact on physical and mental health problems in caregivers [34]. In the current study, most caregivers were patient’s spouses, retired or housewives with an average age of about 63 years and about half of sample were taking care of the PD patients full time, resulting in a high demand of caring. Also, around 12 % of caregivers had been forced to leave their job to take care of PD patient. These results concur with previous studies in Spain and other countries [35, 36] and suggest that family caregivers assume a considerable role in the demands of caring of patients.

In the current study, the related domains of the PDQ-Carer were found to be correlated with all dimensions of the generic SF-36, a widely used health related quality of life in different populations. For all scales, both measures were correlated at significant level, with coefficients values between -0.31 to -0.59 (the negative correlations are due to the fact the measures are scored in opposite directions with 100 being the best score on the SF-36 and the worst on the PDQ-Carer). The personal and social activities, mental health and the anxiety and depression subscales of PDQ-Carer were moderately correlated with the social functioning and general health perceptions of the SF-36. The moderate association found in our study between these measures were similar to those reported by Morley et al., [25]. They found that the PDQ-Carer and SF-36 were significantly correlated, particularly for the social functioning, vitality and mental health SF-36 domains with correlations ranging between -0.68, -0.67, -0.66 respectively. Also, the results of the construct validity of the Spanish version of the PDQ-Carer is comparable to the original English version. These initial findings support the use of the Spanish version of the PDQ-Carer by providing some evidence of the utility of this measure in non-professionals caregivers.

However, the current study has several limitations which require areas for future examination. Firstly, the cross-sectional design of this study provides preliminary evidence of the utility of the PDQ-Carer as a valid and feasible measure for assessing the negative effects on caregiver’s quality of life, but it does not allow to establish temporal consistency of findings across time. Secondly, although participants in our cohort were comprised from different Parkinson’s Associations across Spain, this sample cannot be considered as representative from all the Spanish caregivers since some associations could not be contacted. Consequently, this might limit the generalizability of the findings. Future studies would benefit from the inclusion a large sample size of participants from more diverse settings and regions in Spain. Also, future research should include the use of validated measures assessing the physical stage of the patient’s disease and the examination of caregivers with similar education level. Thirdly, while the focus of the present study was to examine the preliminary findings of Spanish version of the PDQ-Carer in terms of reliability and validity, we did not examine test-rest reliability over time. In addition, future work would benefit from including more sophisticated analysis such as structural equation modelling by considering how some potential moderators such as cognitive deficits, depression or severity of illness, can mediate the impact on caregiver quality of life.

Despite these limitations, this study provides evidence for the use of the PDQ-Carer scale in non-professional caregivers in Spain and to the best of our knowledge is one of the first health related QoL measures designed for caregivers of patients with PD. The use of the PDQ-Carer may have important implications for clinical practice. It may be important for targeting interventions focused on reducing the negative effects on health and social functioning of caregivers as well as to understand the effects of treatment, since those with a poor quality of life may require additional support from clinical and community services.

## Conclusions

﻿Due to the fact the PDQ-Carer is both short and easy to administer, it can be used in a wide range of clinical applications thereby providing important information about health related quality of life problems that need to be addressed in PD caregivers.
